# Substitutional disorder in bis­[(cyanato-κ*O*)/hydroxido(0.5/0.5)](5,10,15,20-tetra­phenyl­porphyrinato-κ^4^ *N*)tin(IV) 

**DOI:** 10.1107/S1600536811021544

**Published:** 2011-06-11

**Authors:** Imen Ben Moussa, Mohamed Salah Belkhiria, Shabir Najmudin, Cecilia Bonifacio, Habib Nasri

**Affiliations:** a Département de Chimie, Faculté des Sciences de Monastir, Université de Monastir, Avenue de l’Environnement, 5019 Monastir, Tunisia; b Faculdade de Medicina Veterinária, Universidade Técnica de Lisboa, Avenida da Universidade Técnica, 1300-477 Lisboa, Portugal; c Departamento de Química, FCT-UNL, 2829-516 Caparica, Portugal

## Abstract

The title complex, [Sn^IV^(C_44_H_28_N_4_)(CNO)(OH)], exhibits substitutional disorder of the OH^−^ and OCN^−^ axial ligands. Thus, the cyanato-*O* ligand and the hydroxyl group bonded to the central Sn^IV^ atom share statistically the axial position. The Sn^IV^ ion is hexa­coordinated by the four N atoms of the pyrrole rings of the tetra­phenyl­porphyrin (TPP) and the O atoms of the two disordered OCN^−^ and OH^−^ axial ligands. The equatorial tin–pyrrole N atom distance (Sn—N_p_) is 2.100 (2) Å and the axial Sn—O(OCN) or Sn—O(OH) bond length is 2.074 (2) Å.

## Related literature

For a review of porphyrin complexes, see: Scheidt (2000 ▶). For the synthesis of tin(IV) porphyrin species, see: Fallon *et al.* (2002 ▶); Martelli *et al.* (2009 ▶). For comparative bond lengths, see: Allen *et al.* (1987 ▶); Smith *et al.* (1991 ▶). For a description of the Cambridge Structural Database, see: Allen (2002 ▶).
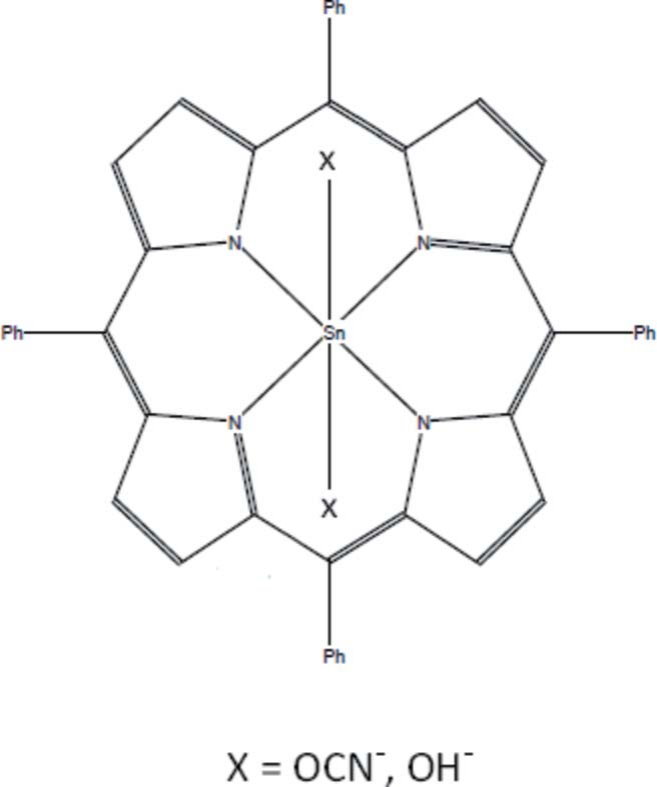


## Experimental

### 

#### Crystal data

[Sn(C_44_H_28_N_4_)(CNO)(OH)]
*M* *_r_* = 790.42
Monoclinic, 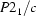

*a* = 11.2943 (6) Å
*b* = 12.6972 (7) Å
*c* = 13.0711 (7) Å
β = 114.251 (2)°
*V* = 1709.06 (16) Å^3^
*Z* = 2
Mo *K*α radiation
μ = 0.80 mm^−1^
*T* = 293 K
0.20 × 0.18 × 0.12 mm

#### Data collection

Bruker APEXII CCD area-detector diffractometer
Absorption correction: multi-scan (*SADABS*; Bruker, 2007 ▶) *T*_min_ = 0.870, *T*_max_ = 0.954
27811 measured reflections
5968 independent reflections
5241 reflections with *I* > 2σ(*I*)
*R*_int_ = 0.028

#### Refinement

*R*[*F* ^2^ > 2σ(*F* ^2^)] = 0.039
*wR*(*F* ^2^) = 0.097
*S* = 1.13
5968 reflections
250 parameters
H-atom parameters constrained
Δρ_max_ = 0.73 e Å^−3^
Δρ_min_ = −1.34 e Å^−3^

### 

Data collection: *APEX2* (Bruker, 2007 ▶); cell refinement: *SAINT* (Bruker, 2007 ▶); data reduction: *SAINT*; program(s) used to solve structure: *SIR2004* (Burla *et al.*, 2005 ▶); program(s) used to refine structure: *SHELXL97* (Sheldrick, 2008 ▶); molecular graphics: *ORTEPIII* (Burnett & Johnson, 1996 ▶) and *ORTEP-3 for Windows* (Farrugia, 1997 ▶); software used to prepare material for publication: *publCIF* (Westrip 2010 ▶).

## Supplementary Material

Crystal structure: contains datablock(s) I, global. DOI: 10.1107/S1600536811021544/dn2696sup1.cif

Structure factors: contains datablock(s) I. DOI: 10.1107/S1600536811021544/dn2696Isup2.hkl

Additional supplementary materials:  crystallographic information; 3D view; checkCIF report

## Figures and Tables

**Fig. 1.**
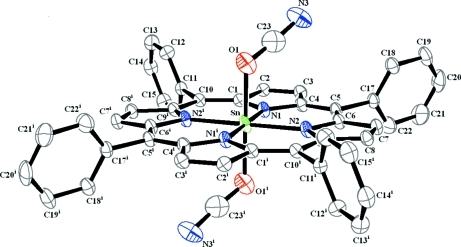
A view of the structure of the complex [SnIV(C44H28N4)(OCN)(OH)] showing the atom numbering scheme. Displacement ellipsoids are drawn at 30%. The O atoms of the hydroxo axial ligands and the H atoms have been omitted for clarity. [Symmetry code: (i) -x + 1,-y, -z + 1]

**Fig. 2.**
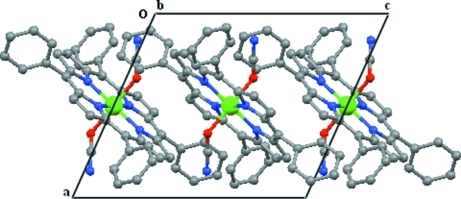
A unit cell packing of the title complex viewed down the b axis. H atoms have been omitted for clarity.

## supplementary crystallographic information

### Comment

The search in the Cambridge Crystallographic Database (version 5.32 with addenda up to November 26, 2010; Allen, 2002) shows that the majority of the reported X-ray molecular structures of porphyrin tin(IV) complexes are hexa-coordinated type [Sn^IV^(Porph)(*X*)_2_] for which *X* is an anionic unidentate ligand bonded to the tin(IV) ion through the oxygen atom. To the best of our knowledge, there is no X-ray molecular structure of a tin(IV) cyanato-*O* porphyrin species reported in the literature.

The bis{(cyanato-*O*)/(hydroxo)(0.5/0.5) }(5,10,15,20-tetraphenylporphyrin)tin(IV) complex [Sn^IV^(C_44_H_28_N_4_)(OCN)(OH)] exhibits substitutional disorder of both thiocyanato-*O* and hydroxo ligands with equal occupancy factor of 0.5. The tin atom which is octahedrally coordinated lies on an inversion center (Fig. 1).

The equatorial tin–pyrrole N atom distance (Sn—N_p_) is 2.100 (2) Å which is normal for tin(IV) porphyrin species. The Sn—O(OH) distance is 2.088 (6) Å which is longer than the one of the related species [Sn^IV^(TPP)(OH)_2_] (2.023 (4) Å) (Smith *et al.*, 1991). The Sn—O(OCN) bond lengh value is 2.059 (8) Å which is close to those of related porphyrin species, i.e, for [Sn^IV^(TTP)(OC_6_H_5_)_2_] (TTP is the *meso*-tetrakis(*p*-tolyl)porphyrin) (Fallon *et al.*, 2002) the Sn—O(OPh) distance is 2.055 (2) Å.

There are no intermolecular or intramolecular hydrogen bonds in the structure of (I). The packing diagram for (I) (Fig.2) is simple; there is no evidence for intermolecular π -π bonding between the faces of the porphyrin cores in compound (I). The absence of the π-π interactions results mainly in the steric restrictions requirements of the phenyl groups that determine the packing environment.

### Experimental

The reaction of the [Sn^IV^(TPP)(OH)_2_] complex (30 mg, 0.037 mmol) (Martelli *et al.*, 2009) with an excess of sodium cyanate, NaOCN (85 mg, 1.31 mmol) and 18-crown-6 (90 mg, 0.34 mmol) in dichloromethane (4 ml) give a pink-violet solution. Crystals of the title complex were obtained by diffusion of ether through the dichloromethane solution.

### Refinement

The position of the O atoms of the NCO and OH couldn't be separated and were located on the same site using the EXYZ and EADP commands within SHELXL-97 (Sheldrick, 2008).

Hydrogen atoms were placed using assumed geometrically idealized positions (C—H aromatic = 0.95 Å) and constrained to ride on their parent atoms, with *U*(H) = 1.2*Ueq*(C). The H atom pertaining to the hydroxo ligand could not be found in a difference Fourier but was introduced in idealized position and treated as riding with *U*(H) = 1.5*Ueq*(O)

### Crystal data

**Table:**

[Sn(C_44_H_28_N_4_)(CNO)(OH)]	*F*(000) = 800
*M**_r_* = 790.42	*D*_x_ = 1.536 Mg m^−^^3^
Monoclinic, *P*2_1_/*c*	Mo *K*α radiation, λ = 0.71073 Å
Hall symbol: -P 2ybc	Cell parameters from 27811 reflections
*a* = 11.2943 (6) Å	θ = 2.6–32.2°
*b* = 12.6972 (7) Å	µ = 0.80 mm^−^^1^
*c* = 13.0711 (7) Å	*T* = 293 K
β = 114.251 (2)°	Prism, purple
*V* = 1709.06 (16) Å^3^	0.20 × 0.18 × 0.12 mm
*Z* = 2	

### Data collection

**Table:**

Bruker APEXII CCD area-detector diffractometer	5968 independent reflections
Radiation source: fine-focus sealed tube	5241 reflections with *I* > 2σ(*I*)
graphite	*R*_int_ = 0.028
φ and ω scans	θ_max_ = 32.2°, θ_min_ = 2.6°
Absorption correction: multi-scan (*SADABS*; Bruker, 2007)	*h* = −16→15
*T*_min_ = 0.870, *T*_max_ = 0.954	*k* = 0→18
27811 measured reflections	*l* = 0→19

### Refinement

**Table:**

Refinement on *F*^2^	Primary atom site location: structure-invariant direct methods
Least-squares matrix: full	Secondary atom site location: difference Fourier map
*R*[*F*^2^ > 2σ(*F*^2^)] = 0.039	Hydrogen site location: inferred from neighbouring sites
*wR*(*F*^2^) = 0.097	H-atom parameters constrained
*S* = 1.13	*w* = 1/[σ^2^(*F*_o_^2^) + (0.0357*P*)^2^ + 1.5751*P*] where *P* = (*F*_o_^2^ + 2*F*_c_^2^)/3
5968 reflections	(Δ/σ)_max_ < 0.001
250 parameters	Δρ_max_ = 0.73 e Å^−^^3^
0 restraints	Δρ_min_ = −1.34 e Å^−^^3^

### Special details

**Table:**

Geometry. All esds (except the esd in the dihedral angle between two l.s. planes) are estimated using the full covariance matrix. The cell esds are taken into account individually in the estimation of esds in distances, angles and torsion angles; correlations between esds in cell parameters are only used when they are defined by crystal symmetry. An approximate (isotropic) treatment of cell esds is used for estimating esds involving l.s. planes.
Refinement. Refinement of *F*^2^ against ALL reflections. The weighted *R*-factor *wR* and goodness of fit *S* are based on *F*^2^, conventional *R*-factors *R* are based on *F*, with *F* set to zero for negative *F*^2^. The threshold expression of *F*^2^ > σ(*F*^2^) is used only for calculating *R*-factors(gt) *etc*. and is not relevant to the choice of reflections for refinement. *R*-factors based on *F*^2^ are statistically about twice as large as those based on *F*, and *R*- factors based on ALL data will be even larger.

### Fractional atomic coordinates and isotropic or equivalent isotropic displacement parameters (Å^2^)

**Table:**

	*x*	*y*	*z*	*U*_iso_*/*U*_eq_	Occ. (<1)
Sn1	0.5000	0.0000	0.5000	0.02969 (7)	
N2	0.50052 (16)	0.15106 (14)	0.43522 (13)	0.0276 (3)	
N1	0.63300 (16)	0.04991 (14)	0.65913 (13)	0.0269 (3)	
O1	0.65060 (16)	−0.03859 (15)	0.45502 (15)	0.0389 (4)	0.50
N3	0.8546 (4)	0.0655 (5)	0.5244 (4)	0.0513 (12)	0.50
C23	0.7509 (6)	0.0155 (5)	0.4921 (5)	0.0479 (13)	0.50
O2	0.65060 (16)	−0.03859 (15)	0.45502 (15)	0.0389 (4)	0.50
H2A	0.6917	−0.0883	0.4933	0.058*	0.50
C1	0.68716 (18)	−0.01306 (15)	0.75191 (15)	0.0245 (3)	
C2	0.78155 (19)	0.04888 (16)	0.84031 (15)	0.0259 (4)	
H2	0.8319	0.0262	0.9130	0.031*	
C3	0.78367 (19)	0.14687 (17)	0.79813 (16)	0.0274 (4)	
H3	0.8360	0.2030	0.8365	0.033*	
C4	0.68969 (18)	0.14729 (16)	0.68298 (16)	0.0262 (4)	
C5	0.66400 (19)	0.23239 (16)	0.60800 (16)	0.0266 (4)	
C6	0.57669 (19)	0.23375 (16)	0.49389 (16)	0.0268 (4)	
C7	0.55237 (19)	0.32147 (16)	0.41809 (17)	0.0284 (4)	
H7	0.5903	0.3878	0.4360	0.034*	
C8	0.46382 (19)	0.29004 (16)	0.31546 (16)	0.0279 (4)	
H8	0.4310	0.3306	0.2505	0.033*	
C9	0.43046 (18)	0.18217 (16)	0.32611 (15)	0.0253 (4)	
C10	0.65856 (18)	−0.11936 (16)	0.75997 (15)	0.0247 (3)	
C11	0.73248 (19)	−0.17185 (16)	0.87048 (15)	0.0251 (3)	
C12	0.8373 (2)	−0.23659 (18)	0.88507 (17)	0.0314 (4)	
H12	0.8602	−0.2483	0.8253	0.038*	
C13	0.9080 (2)	−0.2840 (2)	0.98771 (19)	0.0376 (5)	
H13	0.9778	−0.3274	0.9964	0.045*	
C14	0.8756 (2)	−0.2673 (2)	1.07638 (18)	0.0387 (5)	
H14	0.9238	−0.2986	1.1454	0.046*	
C15	0.7702 (3)	−0.2033 (2)	1.06295 (18)	0.0393 (5)	
H15	0.7475	−0.1924	1.1229	0.047*	
C16	0.6988 (2)	−0.15561 (19)	0.96030 (17)	0.0334 (4)	
H16	0.6285	−0.1128	0.9516	0.040*	
C17	0.74361 (19)	0.32920 (16)	0.65294 (17)	0.0280 (4)	
C18	0.8459 (2)	0.35026 (19)	0.62357 (18)	0.0341 (4)	
H18	0.8621	0.3061	0.5740	0.041*	
C19	0.9245 (2)	0.4378 (2)	0.6684 (2)	0.0448 (6)	
H19	0.9926	0.4522	0.6481	0.054*	
C20	0.9024 (3)	0.5030 (2)	0.7422 (3)	0.0563 (8)	
H20	0.9560	0.5608	0.7725	0.068*	
C21	0.8015 (4)	0.4829 (2)	0.7712 (3)	0.0622 (9)	
H21	0.7861	0.5275	0.8208	0.075*	
C22	0.7216 (3)	0.3962 (2)	0.7270 (3)	0.0490 (6)	
H22	0.6530	0.3831	0.7472	0.059*	

### Atomic displacement parameters (Å^2^)

**Table:**

	*U*^11^	*U*^22^	*U*^33^	*U*^12^	*U*^13^	*U*^23^
Sn1	0.03022 (10)	0.02873 (10)	0.02008 (9)	−0.01209 (8)	0.00016 (7)	0.00446 (7)
N2	0.0283 (8)	0.0275 (8)	0.0206 (7)	−0.0088 (6)	0.0037 (6)	0.0030 (6)
N1	0.0266 (7)	0.0268 (8)	0.0208 (7)	−0.0068 (6)	0.0031 (6)	0.0026 (6)
O1	0.0350 (8)	0.0416 (9)	0.0396 (9)	0.0014 (7)	0.0149 (7)	0.0072 (7)
N3	0.037 (2)	0.076 (4)	0.044 (2)	−0.013 (2)	0.0184 (19)	−0.005 (2)
C23	0.048 (3)	0.055 (3)	0.040 (3)	0.001 (2)	0.017 (2)	0.005 (2)
O2	0.0350 (8)	0.0416 (9)	0.0396 (9)	0.0014 (7)	0.0149 (7)	0.0072 (7)
C1	0.0231 (8)	0.0279 (9)	0.0195 (7)	−0.0017 (7)	0.0057 (6)	0.0009 (6)
C2	0.0249 (8)	0.0288 (9)	0.0202 (7)	−0.0008 (7)	0.0055 (6)	−0.0021 (7)
C3	0.0247 (8)	0.0305 (10)	0.0224 (8)	−0.0054 (7)	0.0049 (7)	−0.0033 (7)
C4	0.0240 (8)	0.0283 (9)	0.0221 (8)	−0.0063 (7)	0.0054 (6)	−0.0011 (7)
C5	0.0257 (8)	0.0256 (9)	0.0248 (8)	−0.0073 (7)	0.0066 (7)	−0.0009 (7)
C6	0.0257 (8)	0.0264 (9)	0.0241 (8)	−0.0074 (7)	0.0060 (7)	0.0018 (7)
C7	0.0287 (9)	0.0254 (9)	0.0280 (9)	−0.0068 (7)	0.0085 (7)	0.0032 (7)
C8	0.0283 (9)	0.0278 (9)	0.0254 (8)	−0.0035 (7)	0.0088 (7)	0.0053 (7)
C9	0.0247 (8)	0.0268 (9)	0.0217 (7)	−0.0038 (7)	0.0068 (6)	0.0031 (6)
C10	0.0237 (8)	0.0288 (9)	0.0196 (7)	−0.0027 (7)	0.0070 (6)	0.0020 (6)
C11	0.0268 (8)	0.0247 (8)	0.0199 (7)	−0.0035 (7)	0.0056 (6)	0.0005 (6)
C12	0.0309 (9)	0.0356 (11)	0.0238 (8)	0.0024 (8)	0.0072 (7)	−0.0005 (7)
C13	0.0311 (10)	0.0377 (12)	0.0327 (10)	0.0004 (9)	0.0017 (8)	0.0033 (9)
C14	0.0376 (11)	0.0405 (12)	0.0259 (9)	−0.0074 (10)	0.0008 (8)	0.0086 (8)
C15	0.0485 (13)	0.0441 (13)	0.0249 (9)	−0.0062 (11)	0.0148 (9)	0.0042 (9)
C16	0.0388 (11)	0.0375 (11)	0.0264 (9)	0.0011 (9)	0.0159 (8)	0.0033 (8)
C17	0.0271 (8)	0.0241 (9)	0.0266 (8)	−0.0052 (7)	0.0049 (7)	0.0009 (7)
C18	0.0323 (10)	0.0344 (11)	0.0311 (10)	−0.0076 (8)	0.0084 (8)	0.0010 (8)
C19	0.0340 (11)	0.0420 (13)	0.0462 (13)	−0.0153 (10)	0.0042 (10)	0.0105 (11)
C20	0.0528 (16)	0.0293 (12)	0.0615 (18)	−0.0169 (11)	−0.0019 (13)	−0.0039 (12)
C21	0.067 (2)	0.0404 (16)	0.075 (2)	−0.0133 (14)	0.0255 (18)	−0.0291 (15)
C22	0.0483 (14)	0.0454 (15)	0.0580 (16)	−0.0131 (12)	0.0265 (13)	−0.0187 (12)

### Geometric parameters (Å, °)

**Table:**

Sn1—O2^i^	2.0737 (18)	C8—C9	1.442 (3)
Sn1—O1^i^	2.0737 (18)	C8—H8	0.9300
Sn1—O1	2.0737 (18)	C9—C10^i^	1.408 (3)
Sn1—N2^i^	2.0976 (17)	C10—C9^i^	1.408 (3)
Sn1—N2	2.0976 (17)	C10—C11	1.496 (3)
Sn1—N1	2.1018 (16)	C11—C12	1.388 (3)
Sn1—N1^i^	2.1018 (16)	C11—C16	1.390 (3)
N2—C6	1.374 (2)	C12—C13	1.386 (3)
N2—C9	1.375 (2)	C12—H12	0.9300
N1—C4	1.368 (3)	C13—C14	1.368 (4)
N1—C1	1.369 (2)	C13—H13	0.9300
O1—C23	1.241 (6)	C14—C15	1.391 (4)
O1—H2A	0.8202	C14—H14	0.9300
N3—C23	1.244 (7)	C15—C16	1.389 (3)
C23—H2A	1.4810	C15—H15	0.9300
C1—C10	1.402 (3)	C16—H16	0.9300
C1—C2	1.442 (3)	C17—C18	1.385 (3)
C2—C3	1.365 (3)	C17—C22	1.386 (3)
C2—H2	0.9300	C18—C19	1.393 (3)
C3—C4	1.442 (3)	C18—H18	0.9300
C3—H3	0.9300	C19—C20	1.370 (5)
C4—C5	1.407 (3)	C19—H19	0.9300
C5—C6	1.410 (3)	C20—C21	1.364 (5)
C5—C17	1.494 (3)	C20—H20	0.9300
C6—C7	1.440 (3)	C21—C22	1.390 (4)
C7—C8	1.363 (3)	C21—H21	0.9300
C7—H7	0.9300	C22—H22	0.9300
			
O2^i^—Sn1—O1^i^	0.00 (4)	C5—C6—C7	126.15 (18)
O2^i^—Sn1—O1	180.0	C8—C7—C6	107.83 (17)
O1^i^—Sn1—O1	180.0	C8—C7—H7	126.1
O2^i^—Sn1—N2^i^	87.88 (7)	C6—C7—H7	126.1
O1^i^—Sn1—N2^i^	87.88 (7)	C7—C8—C9	107.36 (17)
O1—Sn1—N2^i^	92.12 (7)	C7—C8—H8	126.3
O2^i^—Sn1—N2	92.12 (7)	C9—C8—H8	126.3
O1^i^—Sn1—N2	92.12 (7)	N2—C9—C10^i^	125.74 (18)
O1—Sn1—N2	87.88 (7)	N2—C9—C8	108.13 (16)
N2^i^—Sn1—N2	180.0	C10^i^—C9—C8	126.13 (17)
O2^i^—Sn1—N1	89.10 (7)	C1—C10—C9^i^	126.59 (17)
O1^i^—Sn1—N1	89.10 (7)	C1—C10—C11	116.56 (16)
O1—Sn1—N1	90.90 (7)	C9^i^—C10—C11	116.83 (17)
N2^i^—Sn1—N1	89.75 (6)	C12—C11—C16	119.03 (18)
N2—Sn1—N1	90.25 (6)	C12—C11—C10	120.18 (18)
O2^i^—Sn1—N1^i^	90.90 (7)	C16—C11—C10	120.79 (19)
O1^i^—Sn1—N1^i^	90.90 (7)	C13—C12—C11	120.7 (2)
O1—Sn1—N1^i^	89.10 (7)	C13—C12—H12	119.7
N2^i^—Sn1—N1^i^	90.25 (6)	C11—C12—H12	119.7
N2—Sn1—N1^i^	89.75 (6)	C14—C13—C12	120.3 (2)
N1—Sn1—N1^i^	180.0	C14—C13—H13	119.9
C6—N2—C9	108.73 (16)	C12—C13—H13	119.9
C6—N2—Sn1	125.33 (13)	C13—C14—C15	119.8 (2)
C9—N2—Sn1	125.86 (13)	C13—C14—H14	120.1
C4—N1—C1	109.20 (15)	C15—C14—H14	120.1
C4—N1—Sn1	125.12 (13)	C16—C15—C14	120.2 (2)
C1—N1—Sn1	125.33 (13)	C16—C15—H15	119.9
C23—O1—Sn1	118.8 (3)	C14—C15—H15	119.9
C23—O1—H2A	89.5	C15—C16—C11	120.0 (2)
Sn1—O1—H2A	109.4	C15—C16—H16	120.0
O1—C23—N3	175.3 (6)	C11—C16—H16	120.0
O1—C23—H2A	33.6	C18—C17—C22	119.0 (2)
N3—C23—H2A	144.8	C18—C17—C5	119.1 (2)
N1—C1—C10	126.57 (17)	C22—C17—C5	121.9 (2)
N1—C1—C2	107.80 (17)	C17—C18—C19	119.8 (2)
C10—C1—C2	125.62 (17)	C17—C18—H18	120.1
C3—C2—C1	107.66 (16)	C19—C18—H18	120.1
C3—C2—H2	126.2	C20—C19—C18	120.7 (3)
C1—C2—H2	126.2	C20—C19—H19	119.7
C2—C3—C4	107.30 (17)	C18—C19—H19	119.7
C2—C3—H3	126.3	C21—C20—C19	119.8 (2)
C4—C3—H3	126.3	C21—C20—H20	120.1
N1—C4—C5	126.31 (17)	C19—C20—H20	120.1
N1—C4—C3	108.03 (17)	C20—C21—C22	120.5 (3)
C5—C4—C3	125.64 (18)	C20—C21—H21	119.8
C4—C5—C6	126.92 (18)	C22—C21—H21	119.8
C4—C5—C17	116.08 (16)	C17—C22—C21	120.3 (3)
C6—C5—C17	116.89 (17)	C17—C22—H22	119.9
N2—C6—C5	125.89 (18)	C21—C22—H22	119.9
N2—C6—C7	107.95 (16)		

Symmetry codes: (i) −*x*+1, −*y*, −*z*+1.

## References

[bb1] Allen, F. H. (2002). *Acta Cryst.* B**58**, 380–388. 10.1107/s0108768102003890 12037359

[bb2] Allen, F. H., Kennard, O., Watson, D. G., Brammer, L., Orpen, A. G. & Taylor, R. (1987). *J. Chem. Soc. Perkin Trans. 2*, pp. S1–19.

[bb3] Bruker (2007). *APEX2*, *SAINT* and *SADABS* Bruker AXS Inc., Madison, Wisconsin, USA.

[bb4] Burla, M. C., Caliandro, R., Camalli, M., Carrozzini, B., Cascarano, G. L., De Caro, L., Giacovazzo, C., Polidori, G. & Spagna, R. (2005). *J. Appl. Cryst.* **38**, 381–388.

[bb5] Burnett, M. N. & Johnson, C. K. (1996). Report ORNL-6895, Oak Ridge National Laboratory, Tennesse, USA.

[bb6] Fallon, G. D., Lee, M. A.-P., Langford, S. J. & Nichols, P. J. (2002). *Org. Lett.* **4**, 1895–1998. 10.1021/ol025935u 12027641

[bb7] Farrugia, L. J. (1997). *J. Appl. Cryst.* **30**, 565.

[bb8] Martelli, C., Canning, J., Reimers, J. R., Sintic, M., Stocks, D., Khoury, T. & Crossley, M. J. (2009). *J. Am. Chem. Soc.* **131**, 2925–2933. 10.1021/ja8081473 19203267

[bb9] Scheidt, W. R. (2000). *The Porphyrin Handboo*k, Vol. 3, edited by K. M. Kadish, R. M. Smith & R. Guilard, pp. 49–112. San Diego: Academic Press.

[bb10] Sheldrick, G. M. (2008). *Acta Cryst.* A**64**, 112–122. 10.1107/S0108767307043930 18156677

[bb11] Smith, G., Arnold, D. P., Kennard, C. H. L. & Mak, T. C. W. (1991). *Polyhedron*, **10**, 509–516.

[bb12] Westrip, S. P. (2010). *J. Appl. Cryst.* **43**, 920–925.

